# Autoimmune disease and risk of postpartum venous thromboembolism

**DOI:** 10.1016/j.rpth.2023.100091

**Published:** 2023-02-23

**Authors:** Rob F. Walker, Neil A. Zakai, Susan M. Mason, Richard F. MacLehose, Faye L. Norby, Line H. Evensen, Alvaro Alonso, Pamela L. Lutsey

**Affiliations:** 1 School of Public Health, Division of Epidemiology and Community Health, University of Minnesota, Minneapolis, Minnesota, USA; 2 Department of Medicine, Larner College of Medicine at the University of Vermont, Burlington, Vermont, USA; 3 Department of Pathology and Laboratory Medicine, Larner College of Medicine at the University of Vermont, Burlington, Vermont, USA; 4 Department of Cardiology, Smidt Heart Institute, Cedars-Sinai Health System, Los Angeles, California, USA; 5 K.G. Jebsen - Thrombosis Research and Expertise Center, Department of Clinical Medicine, UiT-The Arctic University of Norway, Tromsø, Norway; 6 Department of Epidemiology, Rollins School of Public Health, Emory University, Atlanta, Georgia, USA

**Keywords:** autoimmune diseases, postpartum period, pregnancy, systemic lupus erythematosus, venous thromboembolism

## Abstract

**Background:**

The risk of pregnancy-related mortality in the United States has nearly doubled since 1990, with venous thromboembolism (VTE) accounting for approximately 10% of these deaths.

**Objectives:**

The objective of this study was to assess whether preexisting autoimmune disease is a risk factor for postpartum VTE.

**Methods:**

Using the MarketScan Commercial and Medicare Supplemental administrative databases, a retrospective cohort study analyzed whether postpartum persons with autoimmune disease had a higher risk of postpartum VTE incidence than postpartum persons without autoimmune disease. Using International Classification of Diseases codes, we identified 757,303 individuals of childbearing age who had a valid delivery date with at least 12 weeks of follow-up.

**Results:**

Individuals were, on average, 30.7 years old (SD, 5.4), and 3.7% (*N* = 27,997 of 757,303) of them had evidence of preexisting autoimmune disease. In covariate-adjusted models, postpartum persons with preexisting autoimmune disease had higher rates of postpartum VTE than postpartum persons without autoimmune disease (hazard ratio [HR], 1.33; 95% CI, 1.07-1.64). When analyzed by individual autoimmune disease, those with systemic lupus erythematosus (HR, 2.49; 95% CI, 1.47-4.21) and Crohn’s disease (HR, 2.49; 95% CI, 1.34-4.64) were at an elevated risk of postpartum VTE compared with those without autoimmune disease.

**Conclusion:**

Autoimmune disease was associated with a higher rate of postpartum VTE, with evidence that the association was most pronounced among individuals with systemic lupus erythematosus and Crohn’s disease. These findings suggest that postpartum persons of childbearing age with autoimmune disease may require more monitoring and prophylactic care after delivery to prevent potentially fatal VTE events.

## Essentials

• Pregnancy and autoimmune diseases have inflammatory factors that increase the risk of blood clots.
• We tested whether autoimmune disease was associated with postpartum venous thromboembolism (VTE).
• Postpartum persons with autoimmune disease had a 33% increased risk of postpartum VTE.
• Postpartum persons with Crohn’s disease and systemic lupus erythematosus had the highest VTE risk.

## 1 Introduction

The risk of pregnancy-related mortality has nearly doubled in the United States in the past 30 years, with 20.1 deaths per 100,000 live births reported in 2019 [1,2]. Venous thromboembolism (VTE), which consists of both pulmonary embolism (PE) and deep vein thrombosis (DVT), occurs in approximately 120 of every 100,000 pregnancies [3,4]. PE is responsible for about 9% of pregnancy-associated deaths [1,5]. VTE risk is elevated in pregnancy because of an acquired procoagulant state, mechanical obstruction, and many other factors [6]. These factors increase the risk of VTE antepartum and during the puerperium [7,8]. In the puerperium, VTE risk is highest during the first week after delivery (∼90 per 100,000), declines to ∼25 per 100,000 in the second week, and is near the baseline levels by around the 12th week [9]. Factors such as smoking and obesity, as well as certain obstetric procedures and complications (eg, cesarean delivery, obstetrical hemorrhage, or preeclampsia), confer an increased risk for VTE [[9], [10], [11]]. For pregnant persons perceived to be at particularly high VTE risk, clinical practice guidelines from the American Hematologic Society and several obstetric societies recommend prophylactic measures such as administration of low–molecular-weight heparin during and after pregnancy [[12], [13], [14], [15]]. Pregnancy-related VTE is a significant source of morbidity and mortality, and identification of novel risk factors for pregnancy-associated VTE remains a priority to appropriately target prophylactic measures and thus reduce complications due to pregnancy-associated VTE.

Several autoimmune diseases, such as rheumatoid arthritis (RA), systemic lupus erythematosus (SLE), and inflammatory bowel diseases (IBDs), increase the risk of VTE in the nonpregnant population [[16], [17], [18], [19]]. For example, patients with SLE have up to a 26% risk of experiencing a thrombosis event throughout their disease course [19,20]. These autoimmune diseases are believed to increase the VTE risk by upregulating the body’s procoagulants while simultaneously decreasing the physiological anticoagulants by systemic inflammation. A high prevalence of antiphospholipid antibodies is common in patients with autoimmune disease, leading to an increased risk of antiphospholipid antibody syndrome, which targets phospholipid-protein complexes that are known to increase the risk of VTE [20]. Given that pregnancy itself is a procoagulant state [8], autoimmune diseases may exacerbate the already increased risk during and immediately after pregnancy.

Few studies have estimated the association of autoimmune disease with the risk of pregnancy-associated VTE. A barrier to evaluating this association is the relative rarity of both conditions. To minimize this limitation, we constructed a retrospective cohort using data from a large administrative database composed of medical claims from the employer-provided health insurance. We tested whether the hypothesis that autoimmune disease is associated with an increased risk of VTE in the 12 weeks after delivery. We also evaluated the association of several specific autoimmune conditions with VTE risk, namely thyrotoxicosis, Hashimoto’s thyroiditis, ankylosing spondylitis, psoriasis, RA, SLE, and Crohn’s disease. Among these, we hypothesized that those that were systemic (eg, SLE) would be more strongly associated with postpartum VTE than those that are organ-specific autoimmune diseases (eg, thyrotoxicosis or Hashimoto’s thyroiditis).

## 2 Methods

### 2.1 Data source

A retrospective cohort study was constructed using data obtained from the US IBM MarketScan Commercial Claims and Encounter Database (IBM Watson Health) from 2011 to 2018. The database contains individual-level enrolment, inpatient, outpatient, ancillary, and prescription data from healthcare claims provided by US employers, health plans, and hospitals. Data are compliant with the Health Insurance Portability and Accountability Act and are deidentified. This study was deemed exempt by the University of Minnesota Institutional Review Board.

### 2.2 Study design and study population

From MarketScan, we obtained records for a random sample of 1 million individuals with evidence of pregnancy. Using these enrollees, we identified 814,647 persons with an International Classification of Diseases, Ninth Revision, Clinical Modification (ICD-9-CM), or Tenth Revision (ICD-10-CM) diagnosis inpatient code for delivery. To be included as a delivery in this study, pregnancies had to last at least 20 weeks, indicated by International Classification of Diseases (ICD) codes covering natural delivery, Caesarian sections, or extraction by other methodologies (most codes in V27.xx and Z37.xx). Although the face validity of these codes is high, formal validation studies of ICD codes for all deliveries are lacking. However, validation exists for some specific delivery types, and the accuracy of the codes appears reasonable (eg, caesarian section delivery has 98% specificity and 73% sensitivity) [21,22]. The ICD-9/10 diagnosis and procedure codes used to classify delivery are compiled in Supplementary Table 1. After restricting to individuals of common childbearing age (15-45 years) with 3 months of continuous enrolment before their delivery date, we identified 757,303 persons who delivered for the analytic dataset. Only the first birth in the 7-year follow-up period was considered for this analysis. The start of study follow-up was defined as the date of a pregnant person’s first delivery.

### 2.3 Exposure assessment

Twenty-four unique autoimmune diseases were considered for this analysis and are listed with their corresponding ICD-9/10-CM codes in Supplementary Table 2. The validity of ICD codes for identifying autoimmune disease varies by disease but is generally high [23,24]. For example, a systematic review reported ICD-9-CM code 710.0 to have a PPV of 70% to 90% for identifying SLE [23]. Preexisting autoimmune diseases were identified by the presence of 2 or more inpatient and/or 2 or more outpatient ICD codes occurring more than 7 days apart before the first delivery date. Owing to the low prevalence of several autoimmune diseases among persons of childbearing age, only 7 autoimmune diseases were considered for stratified analyses: thyrotoxicosis, Hashimoto’s thyroiditis, ankylosing spondylitis, psoriasis, RA, SLE, and Crohn’s disease.

### 2.4 Outcome assessment

Postpartum VTE events were confined to cases occurring on the date of delivery and 12 weeks thereafter. Incident VTE events were identified by the presence of at least one inpatient ICD-9/10-CM diagnosis code or 2 outpatient diagnosis codes 7 to 185 days apart for VTE. We used an algorithm of ICD-9/10-CM codes and proof of anticoagulation treatment, which had been validated against medical record review, to detect VTE (PPV = 91%) [25]. Owing to the nature of this manuscript, we also added pregnancy-related ICD-9/10-CM codes to our algorithm (all codes are found in Supplementary Table 3). Inpatient and outpatient diagnoses were confirmed with at least one anticoagulant prescription occurring within 31 days of the VTE date. Patients without an anticoagulant prescription were excluded from the analyses.

### 2.5 Covariates

MarketScan demographic, inpatient, outpatient, and pharmacy claim data before the delivery date were used to assess the presence of relevant covariates. Comorbidities and prescription fills were assessed for any relevant ICD-9 or ICD-10 codes occurring at any time before the delivery date. We used ICD-9/10-CM coding algorithms developed by Quan to identify comorbidities [26]. These algorithms were found to outperform the existing comorbidity scores in administrative data [26].

### 2.6 Statistical analysis

Cox regression was used to estimate hazard ratios (HRs) and 95% CIs for the risk of postpartum VTE within 12 weeks of delivery, comparing postpartum persons with preexisting autoimmune disease with those without autoimmune diseases. Two adjusted models were used. Model 1 adjusted for age, hypertension, diabetes (both mellitus and gestational), preeclampsia, multiple gestation, and antepartum hemorrhage. Model 2 included all comorbidities in Model 1 plus prescription variables (immunosuppressant drugs, dexamethasone, oral prednisone, or hydroxychloroquine). Identical analyses also investigated the risk of postpartum VTE within 0 to 6 weeks after delivery and 7 to 12 weeks after delivery to assess whether risk differed by time postpartum. Associations were also evaluated separately for specific autoimmune diseases where enough VTE cases were available to derive meaningful estimates of association. All data management and analyses were performed using SAS version 9.4 (SAS Inc).

## 3 Results

The average age of postpartum persons in the analytic sample was 30.7 years (SD, 5.4 years), and 27,997 of the 757,303 deliveries (3.7%) occurred among those with preexisting autoimmune diseases. The most prevalent 10 autoimmune diseases (Figure A), together with the number of postpartum VTE events per autoimmune disease (Figure B), are shown in the Figure. The most common autoimmune diseases were thyrotoxicosis (24.5%), Hashimoto’s disease (15.8%), ankylosing spondylitis (11.3%), and psoriasis (9.6%). Postpartum persons with preexisting autoimmune disease had higher proportions of comorbidities and pregnancy complications, and they were more likely to be prescribed immunosuppressant drugs, oral prednisone, hydroxychloroquine, and dexamethasone (Table 1).

**Figure:**
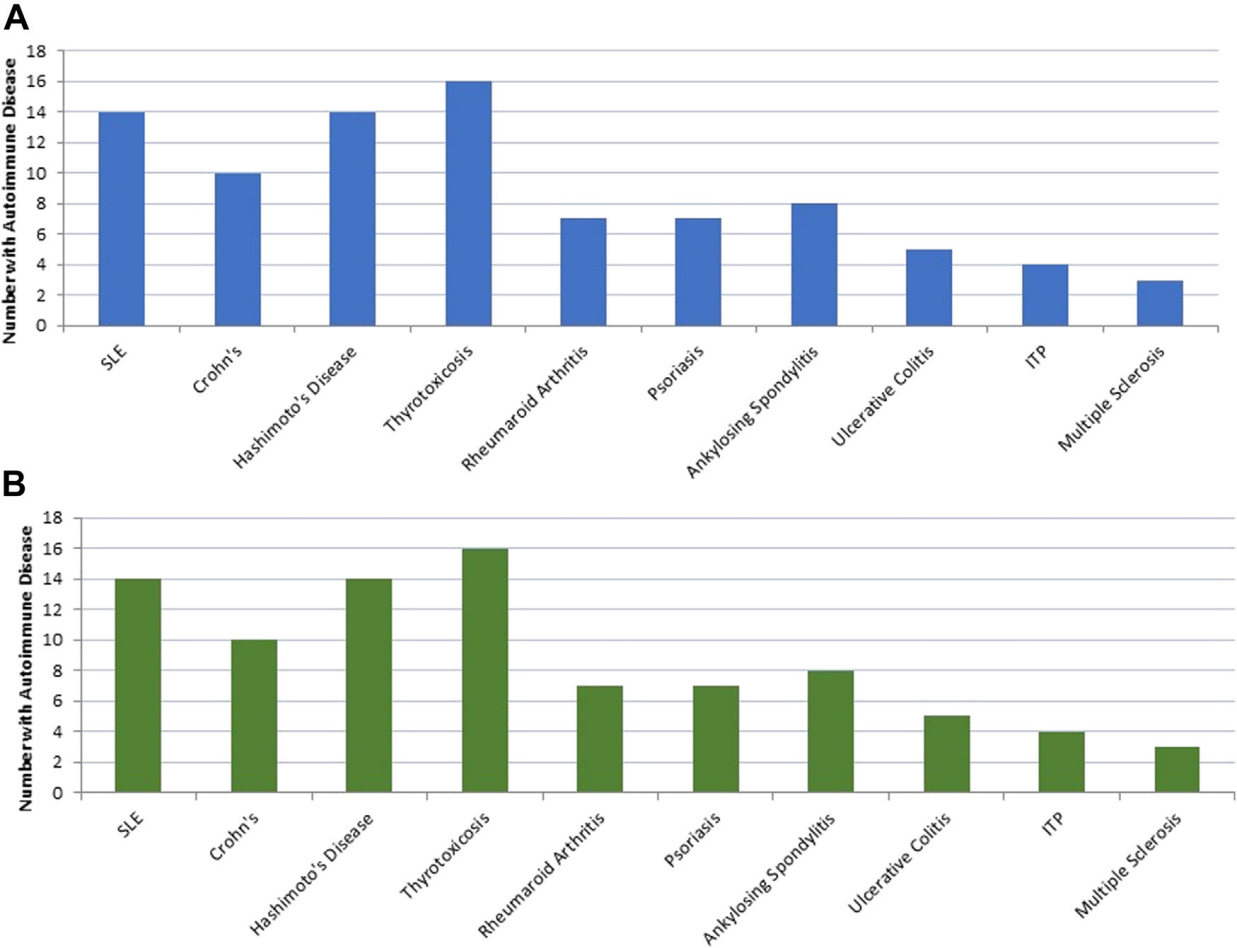
The distribution of (A) preexisting autoimmune disease and (B) number of postpartum venous thromboembolism (VTE) events among diseases in a sample of 27,997 postpartum persons with autoimmune disease who delivered, MarketScan 2011 to 2018. ITP, idiopathic thrombocytopenic purpura; SLE, systemic lupus erythematosus.

**Table 1:** Characteristics of postpartum persons by autoimmune status, MarketScan 2011 to 2018.

Population characteristics	No autoimmune disease	Autoimmune disease
	(*N* = 729,306)	(*N* = 27,997)
Age (y), mean (SD)	32.3 (5.8)	33.1 (5.5)
Person-years follow-up (mean)	1,401,944 (1.9)	51,877 (1.9)
Postpartum VTE	1562	88
Postpartum VTE per 1000 person-y (95% CI)	1.11 (1.06-1.17)	1.70 (1.37-2.08)
Comorbidities, *n* (%)		
Hypertension	29,111 (4.0)	2437 (8.7)
Cancer	4842 (0.7)	655 (2.3)
Diabetes (mellitus and gestational)	108,569 (14.9)	5463 (19.5)
Preeclampsia	50,308 (6.9)	2580 (9.2)
Multiple gestation	23,160 (3.2)	1346 (4.8)
Antepartum hemorrhage	51,951 (7.1)	2800 (10.0)
Chronic pulmonary disease	55,062 (7.6)	3834 (13.7)
Depression	56,210 (7.7)	4237 (15.1)
Medications, *n* (%)		
Immunosuppressants	318 (0.04)	1552 (5.5)
Hydroxychloroquine	253 (0.03)	1403 (5.0)
Oral prednisone	41,679 (5.7)	5912 (21.1)
Dexamethasone	5446 (0.8)	565 (2.0)

VTE, venous thromboembolism.

There were 1650 postpartum VTE events in the study population; among them, 1470 (89.1%) occurred within 0 to 6 weeks after delivery, and 88 (5.3%) occurred in persons with preexisting autoimmune diseases. The Figure shows the number of VTE events stratified by specific autoimmune disease conditions that were the most common. In both the crude and adjusted models, postpartum persons with any autoimmune disease had a higher rate of postpartum rate of VTE (crude HR, 1.47; 95% CI, 1.19, 1.82; Model 1 HR, 1.33; 95% CI, 1.07, 1.64; Model 2 HR, 1.22; 95% CI, 0.97, 1.85) relative to those without (Table 2). When stratifying by time since delivery, although not statistically significant, there was a signal that postpartum persons with autoimmune disease may have slightly higher rates of postpartum VTE 0 to 6 weeks after delivery (fully adjusted HR, 1.16; 95% CI, 0.90, 1.49). Postpartum persons with autoimmune disease did have higher rates of postpartum VTE 7 to 12 weeks after delivery (fully adjusted HR, 1.91; 95% CI, 1.03, 3.54) compared with those without autoimmune diseases. However, the effect estimates contained wide CIs for the latter comparison, with only 11 postpartum VTE events among autoimmune disease occurring. When evaluating the individual autoimmune diseases separately, the rate of postpartum VTE was almost 2 times higher among postpartum persons with SLE (HR, 1.90; 95% CI, 0.98, 3.68) and 2.5 times higher among postpartum persons with Crohn’s disease (HR, 2.45; 95% CI, 1.27, 4.72) relative to postpartum persons without these conditions; however, both of these results are imprecise. Effect estimates for postpartum VTE were close to the null hypothesis among those with Hashimoto’s thyroiditis, thyrotoxicosis, ankylosing spondylitis, psoriasis, and RA, often with very wide CIs owing to the small sample sizes.

**Table 2:** Association of autoimmune disease and risk of postpartum venous thromboembolism by autoimmune disease category, MarketScan 2011 to 2018.

Exposure	Total *N*	Postpartum VTEs	Crude model HR (95% CI)	Adjusted Model 1 HR (95% CI)^a^	Adjusted Model 2 HR (95% CI)^b^
All autoimmune disease	27,997	88	1.47 (1.19-1.82)	1.33 (1.07-1.64)	1.22 (0.97-1.55)
Specific autoimmune disease diagnosis					
Systemic lupus erythematosus	2151	14	3.03 (1.79-5.13)	2.49 (1.47-4.21)	1.90 (0.98-3.68)
Crohn’s disease	1730	10	2.67 (1.43-4.97)	2.49 (1.34-4.64)	2.45 (1.27-4.72)
Hashimoto’s thyroiditis	4415	14	1.46 (0.87-2.48)	1.30 (0.77-2.20)	1.29 (0.76-2.18)
Thyrotoxicosis	6870	16	1.07 (0.65-1.75)	0.97 (0.59-1.58)	0.96 (0.59-1.57)
Rheumatoid arthritis	2478	7	1.30 (0.62-2.73)	1.09 (0.52-2.29)	0.73 (0.32-1.63)
Psoriasis	2686	7	1.20 (0.57-2.51)	1.08 (0.51-2.26)	1.03 (0.48-2.17)
Ankylosing spondylitis	3153	8	1.17 (0.58-2.34)	1.05 (0.53-2.11)	1.04 (0.52-2.08)

HR, hazard ratio; VTE, venous thromboembolism.

a Analyses adjusted for age, hypertension, diabetes, preeclampsia, multiple gestation, and antepartum hemorrhage.

b Analyses adjusted for variables in Model 1 + immunosuppressant drugs, dexamethasone, oral prednisone, and hydroxychloroquine.

## 4 Discussion

In this commercially insured population of approximately 750,000 postpartum persons who had a delivery after at least 20 weeks of gestation, individuals with autoimmune disease had 1.33 times the rate of VTE in the 12 weeks after delivery compared with individuals without autoimmune disease, after controlling for comorbidities. The association was most pronounced among postpartum persons with SLE and those with Crohn’s disease, where the rate was nearly 2.5 times higher compared with postpartum persons without autoimmune diseases. These findings help clarify the diseases that may increase the risk of postpartum VTE and reveal a high-risk group of pregnant and postpartum persons who may require measures to reduce the morbidity and mortality from VTE.

### 4.1 SLE and postpartum VTE

Systemic autoimmune diseases, such as SLE, are associated with increased inflammation, potentially increasing molecular procoagulant factors that can eventually contribute to VTE [27]. Coupled with pregnancy-associated factors (eg, procoagulant state and stasis), systemic autoimmune diseases may increase the risk of VTE. In the present analysis of MarketScan data, postpartum persons with SLE were at a 2.5-fold greater risk of developing VTE.

Patients with SLE have been shown to have an increased risk of VTE, in both general and peripartum populations [19]. A review by Zöller et al. [20] estimated that 10% to 26% of all SLE patients will experience thrombosis during their life. A study of pregnancy-associated VTE, conducted using the Health Care Cost and Utilization Project, Nationwide Inpatient Sample (HCUP-NIS) in the US, found an elevated risk of pregnancy-associated VTE with SLE (odds ratio [OR], 8.20; 95% CI, 6.20, 10.84) and Crohn’s disease (OR, 2.71; 95% CI, 1.50, 4.90) [28]. An important aspect of interpreting HCUP-NIS data, however, is that the records are based on hospitalization (not participant) and so were someone with SLE and VTE hospitalized multiple times that person would appear multiple times in the database. In a general population study by Yusuf et al. [16], those hospitalized with autoimmune diseases associated with antiphospholipid antibodies (including SLE, RA, autoimmune hemolytic anemia, and immune thrombocytopenic purpura) had an elevated risk of VTE during hospitalization (OR ranging from 1.17 to 1.25), controlling for any surgeries, pregnancy, or delivery. This study did not look at stratified results for pregnancy-related VTE. The results from our analysis of MarketScan data complement the existing literature on SLE and VTE and provide additional evidence of elevated risk for persons during the postpartum period.

### 4.2 Crohn’s disease, IBD, and postpartum VTE

Crohn’s disease generally affects the gastrointestinal system; however, inflammation from Crohn’s disease can cause complications outside of the bowel system that increase the risk of VTE [29]. Patients with IBD, including Crohn’s disease and ulcerative colitis, generally have a well-documented risk profile for VTE in the general (nonperipartum) population. A 2001 study by Bernstein et al*.* [30] showed that IBD patients have a 3-fold to 4-fold increased risk of developing a DVT (incidence rate ratio, 4.7; 95% CI, 3.5, 6.3) or PE (incidence rate ratio, 2.9; 95% CI, 1.8, 4.7). These findings are corroborated by several other studies, and effect estimates were often similar for the other IBDs, such as ulcerative colitis. For instance, a Danish study by Kappelman et al*.* [31] found that even young patients with IBD (ie, those aged 20 years and younger) were at an increased risk of VTE, despite their youth and VTE generally occurring at older ages [32]. Evidence of IBD and VTE risk in the peripartum period is somewhat limited. The HCUP-NIS study, which showed a higher risk of pregnancy-associated VTE with SLE, also reported an elevated risk among pregnant persons with Crohn’s disease (OR, 2.71; 95% CI, 1.50, 4.90) [28]. To our knowledge, the only other study investigating IBD and peripartum VTE was a Danish study by Hansen et al. [33]. This study found that individuals with IBD, compared with those without, were at an elevated risk for postpartum VTE both during pregnancy (relative risk, 1.67; 95% CI, 1.15, 2.41) and in the postpartum period (relative risk, 2.10; 95% CI, 1.33, 3.30). The results were similar when the authors evaluated Crohn’s disease, specifically. In the present MarketScan analysis, Chron’s disease was associated with a 2.5-fold increased risk of VTE. We were not able to evaluate ulcerative colitis specifically, owing to its rarity in our study population. Our findings enhance the existing literature suggesting that IBD, specifically Crohn’s disease, is associated with an increased risk of VTE in the postpartum period.

### 4.3 Additional autoimmune disease and postpartum VTE

Several other autoimmune diseases considered in this analysis did not show a significant association with postpartum VTE events (ie, Hashimoto’s thyroiditis, RA, etc.). In many instances, CIs were wide, although point estimates were close to the null, suggesting that it may have been unlikely to see an effect even in a larger sample. Our study included postpartum persons with any evidence of preexisting autoimmune disease, not only recent hospitalization owing to autoimmune disease, and thus, it is possible that the lack of associations for other autoimmune diseases in our study is due to capturing a less severe distribution of these diseases. In addition, many of these diseases are organ-specific, and hence, they were hypothesized to have a lesser effect on VTE risk.

Postpartum persons with Hashimoto’s thyroiditis, the most common cause of hypothyroidism, had a HR of 1.30 (95% CI, 0.77, 2.20) in the present analysis. Previous research has reported hypothyroidism to be modestly associated with both DVT and PE, agreeing with our effect estimate for this particular autoimmune disease [34]. Thyrotoxicosis can sometimes be difficult to diagnose during pregnancy because of similar physiological and hormonal effects, potentially explaining the high number of postpartum persons diagnosed in the present study (nearly 7000) and our null findings [35]. In the present analysis, approximately 2500 postpartum persons had a diagnosis of RA; however, only 7 postpartum VTE events were measured among those with this condition. In the HCUP-NIS study, RA was associated with pregnancy-related PE (OR, 3.62; 95% CI, 1.50, 8.70) [28]. It is common for pregnant persons with RA to be in a “remission” state of disease during pregnancy, potentially explaining our null association with VTE. However, RA remains a well-known risk factor for VTE, and pregnant persons with this condition should be monitored for risk during pregnancy and the postpartum period [20,36]. Both psoriasis and ankylosing spondylitis, systemic autoimmune diseases, are shown to be associated with VTE in nonpregnant populations [[37], [38], [39]]; however, studies that have investigated these diseases and VTE during pregnancy and the postpartum period are scarce. One Scandinavian study looking at pregnant persons with psoriasis and singleton births found no outstanding relationship between psoriasis and pregnancy-related VTE [40]. We also found a null association among those with psoriasis; however, individuals with psoriasis may have been misclassified because of rashes such as eczema or contact dermatitis being coded as psoriasis. All autoimmune disease subclassifications included in this study population had less than 15 postpartum VTE events during the study period. As such, our findings for these autoimmune diseases must be viewed with caution owing to poor precision.

### 4.4 VTE prophylaxis among peripartum persons with autoimmune disease

There is an overall low absolute risk for a pregnancy-related VTE; however, reducing pregnancy-associated VTE is a public health priority, given the potential for devastating consequences, and the observation that underserved and diverse populations are at an increased risk of VTE [41] and have higher a burden of autoimmune diseases [[42], [43], [44]]. Our findings, from a large real-world sample, provide additional support for the existing clinical practice guidelines from the American College of Obstetricians and Gynecologists, the Society of Obstetricians and Gynecologists of Canada, and the Royal College of Obstetricians and Gynecologists (summarized in Table 3) [[12], [13], [14]]. The Society of Obstetricians and Gynecologists of Canada and Royal College of Obstetricians and Gynecologists recommendations include certain autoimmune diseases as “high-risk” factors to be considered when deciding whether to engage with thromboprophylaxis, whereas the American College of Obstetricians and Gynecologists recommendations have no mention of autoimmune diseases other than genetic thrombophilia factors.

**Table 3:** Summary of thromboprophylaxis recommendations for postpartum venous thromboembolism for postpartum persons with/without autoimmune disease.

Society	Prophylaxis recommended in the postpartum period?	Mention of autoimmune disease in determining the need for prophylaxis?	Agent and dosage of thromboprophylaxis
SOGC [13]	Universal postpartum thromboprophylaxis not recommended, assessments required, and prophylaxis based on the presence of preexisting risk factors deemed high-risk by SOGC	Yes, postpartum thromboprophylaxis should be administered if SLE and/or IBD is coupled with another risk factor contributing to a >1% change in VTE	LMWH is preferred
RCOG [14]	Immediate risk assessment and prophylaxis recommended if a woman has 2 noted risk factors deemed high-risk by the RCOG other than a history of VTE or thrombophilia	Yes, prophylactic thromboprophylaxis recommended if SLE and/or antiphospholipid syndrome is coupled with another preexisting, obstetric, or transient risk factor deemed “high-risk” by RCOG	Prophylactic LMWH for at least 10 d postpartum. For those with a history of VTE and antithrombin deficiency, a higher dose of LMWH for 6 wk or oral anticoagulant is restarted, as soon as possible after delivery
ACOG [15]	Dependent on several clinical scenarios. Pregnant persons with no VTE history simply require surveillance. Those with a history of VTE and/or a high-risk thrombophilia are recommended prophylaxis 4-12 h after delivery for up to 6 wk	None, although inherited genetic thrombophilia is highlighted as a risk factor and may be linked with antiphospholipid syndrome	LMWH (prophylactic, intermediate-dose, or adjusted-dose) or unfractionated heparin (prophylactic or adjusted-dose) depending on the clinical scenario and prevalent risk factors

ACOG, American College of Obstetricians and Gynecologists; IBD, inflammatory bowel diseases; LMWH, low–molecular-weight heparin; RCOG, Royal College of Obstetricians and Gynaecologists; SLE, systemic lupus erythematosus; SOGC, Society of Obstetricians and Gynaecologists of Canada; VTE, venous thromboembolism.

### 4.5 Strengths and limitations

Using a large administrative dataset is a source of both strengths and weaknesses of this manuscript. This study evaluated a broad array of autoimmune diseases and pregnancy-related VTE risks in the United States, enabled by the real-world population of over 750,000 pregnant persons that allowed for the prospective assessment of associations between these relatively rare exposures and VTE. However, precision was at times poor for the analyses of specific autoimmune conditions, resulting in wide CIs. Studies regarding autoimmune disease and VTE during pregnancy are relatively uncommon. The most prominent research regarding autoimmune disease and pregnancy-related thrombosis comes from the HCUP-NIS data, which do not allow for the detailed longitudinal analyses and come with inherent limitations because of the following: a) it includes hospitalization discharge information only and b) the same individual may be included in the analysis more than once, if they have multiple discharges [28]. MarketScan’s inclusion of outpatient data allowed for a more comprehensive evaluation of patient history and establishing a clinically meaningful denominator, and the approach is that of a traditional cohort with each individual included in the analysis only once.

As another limitation, variables in this study were defined using ICD codes from the billing data and are subject to misclassification. However, whenever possible, we used ICD-based definitions that were validated against a medical record review to minimize misclassifications. For example, our VTE definition had a positive predictive value (PPV) of 91% [25]. Nonetheless, misclassification remains a threat to validity. Only the first delivery in the study period was used for the analysis, potentially omitting later pregnancies, deliveries, and potential pregnancy-related VTE events. Related to the VTE outcome, an inherent limitation of MarketScan is the lack of information on out-of-hospital death. Therefore, some fatal postpartum VTE events were almost certainly misclassified. In addition, MarketScan includes only individuals with private insurance. Thus, our results may not be generalizable to the enrollees who have severe autoimmune diseases and are uninsured because they are unable to work. Finally, MarketScan did not include information on some key potential covariates, such as race/ethnicity or socioeconomic status (income, education, etc.). Given the health disparities in both maternal mortality [2] and VTE [41], identifying VTE risk factors among racial/ethnic groups at the highest risk is paramount and a limitation in the manuscript.

## 5 Conclusions

Reducing VTE-related maternal mortality and morbidity in the United States, where VTE complicates 1 of every 1000 pregnancies, is vitally important. The findings in the current study suggest values for increasing VTE awareness among pregnant and postpartum persons with autoimmune conditions particularly—SLE, Crohn’s disease—and their healthcare providers. Prophylactic measures are sometimes recommended for high-risk individuals with SLE during pregnancy and may additionally be beneficial for those with IBD, particularly Crohn’s disease, during pregnancy. Research regarding VTE among pregnant persons with autoimmune disease remains sparse, and targeted studies investigating SLE, Crohn’s disease, and less common autoimmune diseases may highlight areas of improvement for pregnancy-related and postpartum thrombosis outcomes among persons of childbearing age.
