# Supplementary material for: Autoimmune disease and risk of postpartum venous thromboembolism
Source: Res Pract Thromb Haemost. 2023 Feb 23;7(2):100091. doi: 10.1016/j.rpth.2023.100091 (PMC10031534; doi:10.1016/j.rpth.2023.100091)
Supplement: Supplemental Tables 1-3 [file mmc1.docx]

Supplemental Table 1. ICD-9-CM and ICD-10-CM diagnosis and procedure codes used to define pregnancies lasting approximately >=20 weeks.

| **Revision** | **Codes** |
| --- | --- |
| ICD-9-CM | V27, 650, 656.4 |
| ICD-10-CM | Z37, O80, O36.4 |
| ICD-9-CM procedure codes | 72, 72.1, 72.21, 72.29, 72.31, 72.39, 72.4, 72.6, 72.51, 72.52, 72.53, 72.54, 72.71, 72.79, 72.8, 72.9, 73.22, 73.59, 73.6, 74, 74.1, 74.2, 74.4, 74.99 |
| ICD-10-CM procedure codes | 10D00ZZ, 10D07Z, 0W8NXZZ, 10E0XZZ |
| CPT-4 Codes | 59514, 59620 |
| DRG version ≤4 | 370-375 |
| DRG version ≥5 | 765-768, 774, 775 |

Supplemental Table 2. ICD-9-CM and ICD-10-CM diagnosis codes used to define prevalent autoimmune diseases.

| **Condition** | **ICD-9-CM codes** | **ICD-10-CM codes** |
| --- | --- | --- |
| Hashimoto’s thyroiditis | 245.2 | E06.3 |
| Thyrotoxicosis | 242 | E05 |
| Ankylosing spondylitis | 720 | M45 |
| Psoriasis | 696 | L40 |
| Rheumatoid arthritis | 714 | M05, M06 |
| Systematic lupus erythematosus | 710.0, 695.4 | M32.1, M32.8, M32.9 |
| Crohn’s disease | 555 | K50 |
|  |  |  |
| Addison’s disease | 255.4 | E27.1 |
| Antiphospholipid syndrome | 298.81 | D68.61 |
| Behcet’s disease | 136.1 | M35.2 |
| Celiac’s disease | 579.0 | K90.0 |
| Chronic active hepatitis | 571.4 | K73.2 |
| Dermatomyotosis & polymyositis | 710.3, 710.4 | M33 |
| Giant cell arteritis | 446.5 | M31.5, M31.6 |
| Goodpasture’s syndrome | 466.21 | M31.0 |
| Guillain-Barre syndrome | 357.0 | G61.0 |
| Hemolytic anemia | 283.0 | D59.0, D59.1 |
| Idiopathic thrombocytopenia purpura | 287.3 | D69.3 |
| Multiple sclerosis | 340 | G35 |
| Myasthenia gravis | 358.0 | G70.0 |
| Pemphigoid | 694.5 | L12 |
| Pemphigus | 694.4 | L10 |
| Pernicious anemia | 281.0 | D51.0 |
| Polyarteritis nodosa | 446.0 | M30.0 |
| Primary biliary cirrhosis | 571.6 | K74.3 |
| Scleroderma | 710.1 | M34 |
| Sjorgen’s syndrome | 710.2 | M35.0 |
| Ulcerative colitis | 556 | K51 |
| Vasculitis | 447.6 | I77.6 |

Supplemental Table 3. ICD-9-CM and ICD-10-CM codes used to define incident postpartum VTE.

| **Revision** | **VTE codes** |
| --- | --- |
| ICD-9-CM | 415.1x, 451.1x, 453.2, 453.4x, 453.82, 453.83, 453.84, 453.85, 453.86, 453.87, 453.89, 453.9, 671.3, 671.4, 671.5, 671.9, 673.2, 673.3, 673.8 |
| ICD-10-CM | I26.0x, I26.9x, I80.1x, I80.20x, I82.210, I80.22x, I80.23x, I80.29x, I82.40x, I82.41x, I82.42x, I82.43x, I82.44x, I82.49x, I82.4Yx, I82.4Zx, I82.60x, I82.62x, I82.890, I82.A1x, I82.B1x, I82.C1x, O22.3, O22.5, O22.9, O87.1, O87.3, O87.9, O88.2, O88.3, O88.8 |
